# At-home blood self-sampling in rheumatology: a qualitative study with patients and health care professionals

**DOI:** 10.1186/s12913-022-08787-5

**Published:** 2022-12-02

**Authors:** Felix Muehlensiepen, Susann May, Joshua Zarbl, Ekaterina Vogt, Katharina Boy, Martin Heinze, Sebastian Boeltz, Hannah Labinsky, Gerlinde Bendzuck, Marianne Korinth, Corinna Elling-Audersch, Nicolas Vuillerme, Georg Schett, Gerhard Krönke, Johannes Knitza

**Affiliations:** 1 Brandenburg Medical School Theodor Fontane, Center for Health Services Research, Seebad 82/83, Rüdersdorf Bei Berlin, 15562 Rüdersdorf, Germany; 2 Faculty of Health Sciences Brandenburg, Brandenburg Medical School Theodor Fontane, Neuruppin, Germany; 3 AGEIS, Université Grenoble Alpes, Grenoble, France; 4 Department of Internal Medicine 3-Rheumatology and Immunology, Friedrich-Alexander University Erlangen-Nürnberg and Universitätsklinikum Erlangen, Erlangen, Germany; 5 Deutsches Zentrum Für Immuntherapie, Friedrich-Alexander University Erlangen-Nürnberg and Universitätsklinikum Erlangen, Erlangen, Germany; 6 Thermo Fisher Scientific, Freiburg, Germany; 7 Department of Psychiatry and Psychotherapy, Brandenburg Medical School Theodor Fontane, Immanuel Klinik Rüdersdorf, Rüdersdorf, Germany; 8 Deutsche Rheuma-Liga Bundesverband E.V, Bonn, Germany; 9 Institut Universitaire de France, Paris, France; 10 LabCom Telecom4Health, Orange Labs & Univ. Grenoble Alpes, CNRS, Inria, Grenoble INP-UGA, Grenoble, France

**Keywords:** Blood self-sampling, Rheumatology, User experience, Remote Care, Qualitative Research, Telemedicine

## Abstract

**Background:**

The goal of the study was to investigate patients’ with systemic rheumatic diseases and healthcare professionals’ experiences and preferences regarding self-sampling of capillary blood in rheumatology care.

**Methods:**

Patients performed a supervised and consecutive unsupervised capillary blood self-collection using an upper arm based device. Subsequently, patients (*n* = 15) and their attending health care professionals (*n* = 5) participated in an explorative, qualitative study using problem-centered, telephone interviews. Interview data were analyzed using structured qualitative content analysis.

**Results:**

Interviewed patients reported easy application and high usability. Patients and health care professionals alike reported time and cost savings, increased independence and flexibility, improved monitoring and reduction of risk of infection during Covid-19 as benefits. Reported drawbacks include limited blood volume, limited usability in case of functional restrictions, and environmental concerns. Older, immobile patients with long journeys to traditional blood collection sites and young patients with little time to spare for traditional blood collection appointments could be user groups, likely to benefit from self-sampling services.

**Conclusions:**

At-home blood self-sampling could effectively complement current rheumatology telehealth care. Appropriateness and value of this service needs to be carefully discussed with patients on an individual basis.

**Trial Registration:**

WHO International Clinical Trials Registry: DRKS00024925. Registered on 15/04/2021.

**Supplementary Information:**

The online version contains supplementary material available at 10.1186/s12913-022-08787-5.

## Introduction

Rising global burden of systemic rheumatic diseases (SRDs) manifests itself in mortality, disability-adjusted life expectancy [1], reduced quality of life [2] as well as direct and indirect health care costs, including absence from work and productivity [3, 4]. The high demand of rheumatology care is opposed by an inadequate number of rheumatologists [5], resulting in diagnostic delays [6] and often consecutive irreversible damage [7]. Covid-19 additionally aggravated this diagnostic delay [8] and the imminent risk of infection lead to a reduction of face-to-face consultations, shift to remote patient care [9] and self-management of patients [8].

Implementation of patient self-management [10] and telehealth strategies [11] could relieve rheumatology care, by accelerating diagnosis [12–14] and enabling a more flexible, need-based patient monitoring [15–18]. Despite the immense significance of laboratory results in rheumatology and the established role of patient self-sampling in the monitoring of other chronic diseases [19, 20], routine rheumatology care does not encompass patient self-sampling yet. However, a recent survey depicted the willingness of rheumatologists and rheumatic patients to incorporate self-sampling into routine care [21] and first rheumatology-based pilot studies highlighted the potential to monitor drug levels [22] and predict disease flares [23] using traditional finger prick devices. Recent trials proved that new upper-arm-based capillary blood self-collection devices such as the Tasso + and TAP II allow practically painless and reliable automatic collection of capillary blood [24–26]. However, these findings cannot be directly applied to rheumatology care as the studies involved distinct patient populations and laboratory parameters.

In a previous randomized controlled trial [27] we compared the acceptance of traditional finger pricking to an upper-arm based device (Tasso-SST) in patients with rheumatoid arthritis. While experienced pain was similarly low in both groups, the upper-arm group was more likely to recommend self-sampling (net promoter score: + 28% vs. -20%) and more willing to perform blood collection at home (60% vs. 32%).

In a consecutive trial we sought to compare two upper arm devices in a more diverse group of patients with systemic rheumatic diseases (SRD). We included an embedded qualitative study to gain in-depth feedback, as to our knowledge, no qualitative study has yet evaluated patient and care provider’s perceptions of capillary blood self-sampling in rheumatology.

The aim of the present qualitative study was therefore to explore SRD-patients' and health care professionals’ (HCP) experiences and preferences regarding at-home capillary blood self-sampling in rheumatology care:

- How do patients and HCP experience upper arm based capillary blood self-sampling?
- Which benefits and drawbacks exist?
- How do patients and HCP assess the transferability of blood self-sampling to standard rheumatology care?

## Methods

We carried out a prospective study to evaluate the feasibility, accuracy, pain, usability and acceptability regarding self-collection of capillary blood for analysis of autoantibodies and inflammation markers in patients with SRD. The study was prospectively registered on 15/04/2021 (WHO International Clinical Trials Registry: DRKS00024925), approved by the local ethics authorities (Reg no. 320_20B) and designed together with three official patient partners (GB, MK, CE; Deutsche Rheuma-Liga Bundesverband e.V). Patients and HCP provided written informed consent prior to participating in the study.

**Fig. 1:**
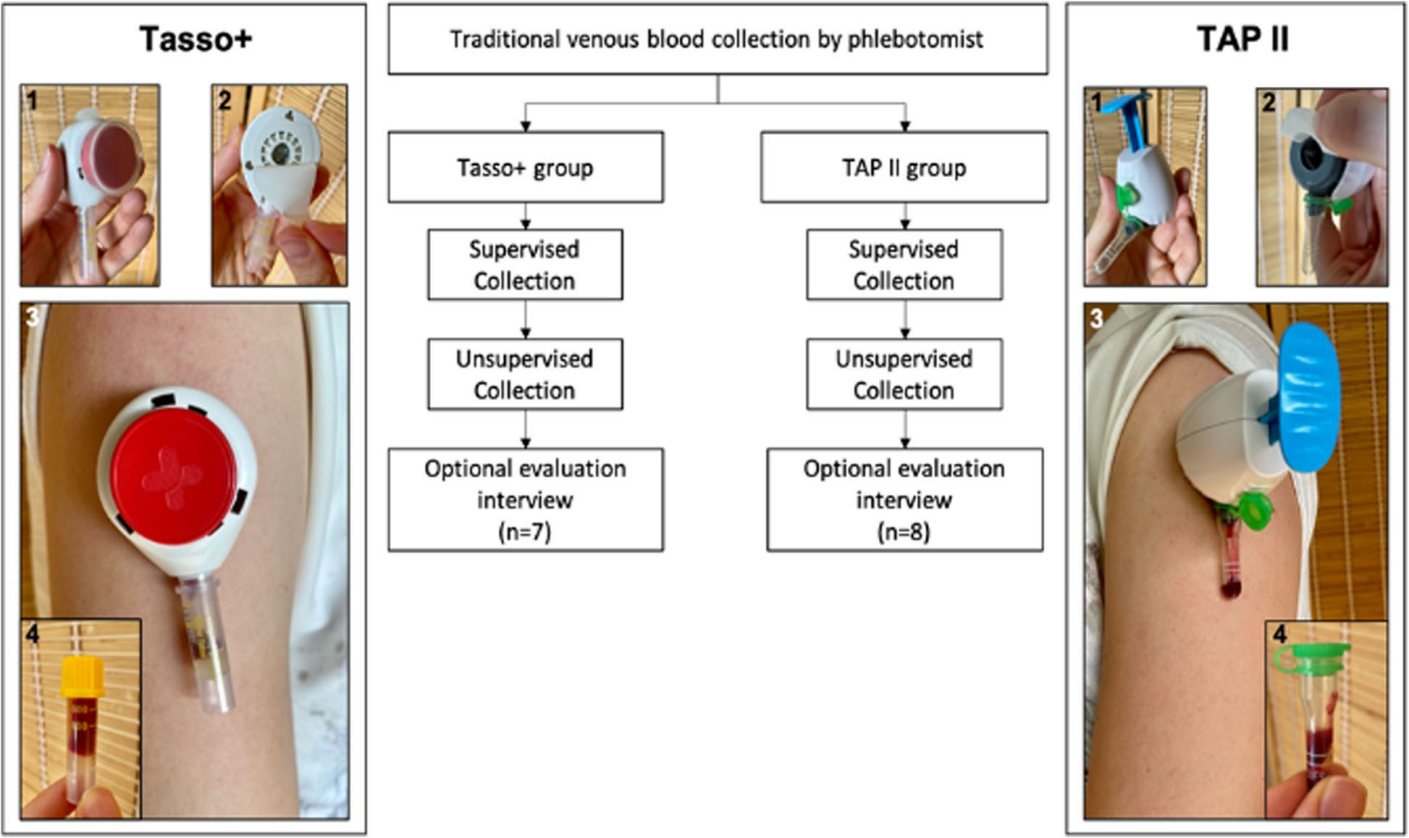
Study flow and main blood self-collection steps

Consecutive patients with a systemic rheumatic disease and previously documented SRD associated autoantibodies were recruited from the in- and outpatient clinics of the Department of Rheumatology at the University Hospital Erlangen. The study team alternately allocated patients to one of two upper-arm based blood collection devices, Tasso + (Tasso Inc., Seattle, WA, USA) or TAP II (YourBio Health, USA), and performed one supervised and one unsupervised blood collection with the respective device (Fig. 1) [28].

Prior to the supervised blood collection patients were given access to written instructions and an instructional video (Tasso + : https://www.tassoinc.com/tasso-plus; TAP II: https://bit.ly/3HCngxK).

One medical student (JZ) supervised all blood collection procedures, ensuring that each step of the self-collection procedure was performed correctly, intervening only in the event of potential harm or failure. Study personnel intervention was registered in 6 of 35 (17%) and 8 of 35 (22%) in the Tasso + and TAP II group, respectively [27]. After the supervised blood collection, patients received blood collections kits and postage packages to independently carry out a second blood collection within one week of the appointment. In contrast to traditional finger-prick devices, both devices are upper-arm-based and allow automatic blood-collection. The self-adhesive devices can be attached to the upper arm and by pressing a button the skin is perforated. Capillary blood is then automatically retrieved in the attached collection tube. After blood collection, the collection tube can be removed from the blood collection device and sent via conventional postage to a laboratory for analysis. To gain in-depth detailed feedback regarding perception of self-sampling of capillary blood in rheumatology care, patients and HCPs were invited to participate in a qualitative interview study. Patients were selected using purposive sampling [29] to include a heterogeneous sample regarding age, disease duration, as well as professional and educational backgrounds. HCPs were selected based on their experience with self-sampling of capillary blood in rheumatology care, as treating physicians or being part of the study team in the prospective study.

Interviews were conducted using a structured interview guide that was developed to specifically elicit the participants’ experiences collecting capillary blood independently and unsupervised. The structured interview guide was developed by two health service researchers (FM, SM), one rheumatologist (JK) and previous discussions with three patient research partners (GB, MK, CEA). The interview guides (Supplemental Material 1 & 2) included the following main topics: feasibility of self-sampling, benefits and drawbacks, and transferability to standard care. Initial exploratory questions were then specified by follow-up questions. FM conducted pilot interviews (Patients 1 & 2 and HCP 1) to test and refine the interview guide. No revisions were necessary. The data of the pilot interviews were thus included in the analysis. Additional sociodemographic data were collected, including gender, age, diagnosis, education and profession. In order to reduce the risk of infection and lower patient burden, the interviews were conducted by FM and SM via telephone. The phone interviews took place between August and December 2021. The interviews were recorded and transcribed verbatim.

The interview data was analyzed based on Kuckartz's structured qualitative content analysis [30] supported by MAXQDA software (Verbi GmbH). Codes and categories were developed inductively, and were then incorporated into the data-driven development of the coding tree (Supplemental Material 3). For interviews with patients and HCP, slightly distinct codes were used to reflect different emphases in the interviews between the participant groups. For instance, patient interviews focused on the self-sampling experiences and patients' perception on its utility, whereas the main focus of HCP interviews was on transfer to standard rheumatology care. To ensure traceability, the coding tree and its application were validated through a member check with interview partners, which involved discussions of the coding tree and coded segments with two patients (Patient 2 & Patient 7) and two HCPs (HCP 1 & HCP 3). Data collection and analysis were performed circular by two researchers (FM, SM). Data collection continued until no substantially new findings emerged and meaning saturation was reached. Meaning saturation was defined as the state when no further dimensions, nuances, or insights can be found on a topic [31]. Finally, the two researchers (FM, SM) independently applied the coding tree to the entire material. Applied codes were exchanged and compared, and inconsistencies were resolved. This manuscript has been compiled in accordance with the Consolidated Criteria for Reporting Qualitative Research (COREQ) (Supplemental Material 4) [32]. For the presentation of the results, representative quotes of the transcripts were selected, translated into English and included into tables (Tables 2, 3 and 4).

## Results

### Participant characteristics

From August to December 2021, we conducted a qualitative interview study on self-sampling of capillary blood with patients (*n* = 15) and HCP (*n* = 5). Mean age of interviewed patients (Table 1) was 56 years (range: 33–73). Eight patients used the TAP-II device and seven patients the Tasso + device. Most interviewed patients were female (14/15; 93%). Interviewed patients suffered from a variety of SRD, including rare disease, with systemic lupus erythematosus being the most frequent diagnosis (*n* = 6). Patients received their diagnosis a mean of 10 years ago (range: 1–34). Patients reported diverse occupational backgrounds. Mean duration of the patient interviews was 17 min (range:10–25). Mean HCP age was 28 years (range: 23–34). Three interview partners were men, two women. All interviewed HCP were entry level doctors (*n* = 3) or medical students (*n* = 2). Mean professional experience of the HCP was 2 years (range: 1–6). Mean duration of the HCP interviews was 19 min (range: 10–29).

**Table 1:** Sample Description

Participant Number	Age	Gender	Device	RMD	Years with diagnosis / Professional experience (HCP)	Profession
Patient 1	52	Female	TAP II	Rheumatoid arthritis	32	Marketing
Patient 2	57	Female	TAP II	Systemic sclerosis	3	Retired (pension for reduced earning capacity)
Patient 3	45	Female	TAP II	Systemic lupus erythematosus	9	Currently no professional activity
Patient 4	57	Female	Tasso +	Sjögren's syndrome	17	Human resources specialist
Patient 5	33	Female	Tasso +	Antiphospholipid syndrome	5	Medical supply store specialist
Patient 6	73	Female	Tasso +	Sjögren's syndrome	11	Assembler
Patient 7	60	Female	Tasso +	Rheumatoid arthritis	14	Radiology assistant
Patient 8	71	Male	TAP II	Granulomatosis with polyangiitis	7	Biologist / pharmaceutical industry
Patient 9	60	Female	TAP II	Systemic lupus erythematosus	34	Currently no professional activity
Patient 10	68	Female	TAP II	Sjögren's syndrome	1	Carpenter
Patient 11	54	Female	TAP II	Systemic lupus erythematosus	1	Nursing care for the elderly
Patient 12	49	Female	Tasso +	Systemic lupus erythematosus	9	Office employee
Patient 13	49	Female	TAP II	Sjögren's syndrome	1	Butcher shop saleswoman
Patient 14	62	Female	Tasso +	Systemic lupus erythematosus	1	Commercial employee
Patient 15	49	Female	Tasso +	Systemic lupus erythematosus	12	Business assistant
HCP 1	26	Male	/	/	1	Medical student
HCP 2	28	Female	/	/	2	Assistant physician
HCP 3	30	Male	/	/	6	Assistant physician
HCP 4	23	Female	/	/	1	Medical student
HCP 5	34	Male	/	/	2	Assistant physician

### Feasibility

Patients were asked to describe their self-sampling experiences and their perception on its utility (Table 2). Overall, most patients reported easy application and high usability, with particular emphasis on the low volume of blood required and the absence of pain. The smooth application was also associated with the clear instructions provided by the study team, including an instruction video and written instructions. Although predominantly positive experiences were reported, interviewees mentioned limitations and negative experiences: Three patients reported adverse consequences of self-sampling. These were persisting wounds and hemorrhage. A recurring aspect reported by patients was the lack of sustainability of self-sampling and the high level of packaging waste associated with the sampling kits. Besides, patients reported that pressing the button to initiate the blood draw as well as removing the device and closing the collection tube were rather challenging tasks, especially with certain co-morbidities and low finger strength. One interviewee was concerned that uncontrolled transport might affect sample quality and test validity. The users' reports did not reveal any differences in feasibility between the two investigated self-sampling devices (Tasso + / TAP II).

**Table 2:** Feasibility

**Description of the self-sampling process**
*“Well, first you should tear open the bag, which contains the device. Then there is also a heat pack, which you have to apply to warm the skin. (…) [A]nd then you need the small bag for disinfection. Before you hold the device or press it, you first have to disinfect the arm and then you activate the device. There is a button and you press it and that is practically the needle where the blood goes in. It's a small container that you have to unscrew beforehand and then you press it so that the blood enters. You have five minutes, a maximum of five minutes, so that the amount of blood- There is a label on it, that's how much blood should be in the small container. Then you can remove it [the collection tube] if you think you have enough blood and then you close it and label it. (…) Then it is packed in a bag and then [put] in a small cardboard box. So you can simply give it to the post office. That would be it.” (Patient 6, Sjögren's syndrome, Tasso* + *: 05:24)*
**Positive experiences**
*“I was totally surprised how easy it was and how painless it was. I think that's great.” (Patient 3:* Systemic lupus erythematosus, *05:48)*
*“Everything worked. I did it all as it was written in the instruction manual. I didn't need any help. It may have taken a little longer because the first time I took a spot in my upper arm where maybe not as many veins go through. I first thought maybe nothing was coming out, but actually blood was still coming out to the extent that it should be and was sufficient for the exam.” (Patient 7, Rheumatoid arthritis, Tasso* + *: 03:49)*
*“Okay. Yes, so first he [the study nurse] showed me the video—he had a video and then on paper, on a poster actually—how you have to do it. That was absolutely understandable. Then I did it right away. (…) I took the blood myself. I practically did the procedure, everything that had to be done. This worked very well.” (Patient 12, Systemic lupus erythematosus, Tasso* + *: 03:00)*
**Negative experiences**
*“For whatever reason… I do not understand it. It started bleeding again after an hour. I do have an autoimmune disease; I have delayed wound healing. But there was definitely a crust for a week afterwards. Didn't hurt though, the blood collection. It is not painful. It's painless.” (Patient 4, Sjögren's syndrome, Tasso* + *: 01:32)*
*“I don't know whether it stays or goes, but I still have these little red dots on my arm, (…) I don't know if these marks will stay or that will go away with time.” (Patient 9, Systemic lupus erythematosus, TAP II: 06:42)*
*“So as I said, with the bruise there, this red squiggle, when you have several on your arm, that really doesn't look nice.” (Patient 1, Rheumatoid arthritis, TAP II: 04:40)*
*“However, I had talked to the study nurse, that the device is not so pleasant for environmental reasons, because it can't be used more than once. And this garbage, which then accumulates every time with such a sampling, well, the manufacturer should change something, so that at least the same person can use the device more than once.” (Patient 1, Rheumatoid arthritis, TAP II: 01:02)*
*“The only thing I had problems with was my Raynaud's syndrome, I have problems with my finger joints to apply the force. So the button was quite difficult for me to push through and that worked, but when I took it off, it [blood] ran out quite difficult… into the collecting tube.” (Patient 2, Systemic sclerosis, TAP II: 07:23)*
*"What bothered me a bit was that after removing the device from the arm, there was a relatively large amount of blood on the arm. What further bothered me was that it takes a certain amount of care or skill to then remove this tube from the device and close it correctly.” Patient 8, Granulomatosis with polyangiitis TAP II: 07:20)*
*“I have to take it to the post office. And the post office is no longer emptied three times a day, but once in the evening at 5 p.m. or 6 p.m., I don't know exactly. And then it goes out the next day. By the time it reaches the institute that evaluates the blood, time has already passed. If it is unfavorable also still in the summertime with high temperatures….” (Patient 10, Sjögren's syndrome, TAP II: 03:53)*

### Transfer to standard rheumatology care

**Fig. 2:**
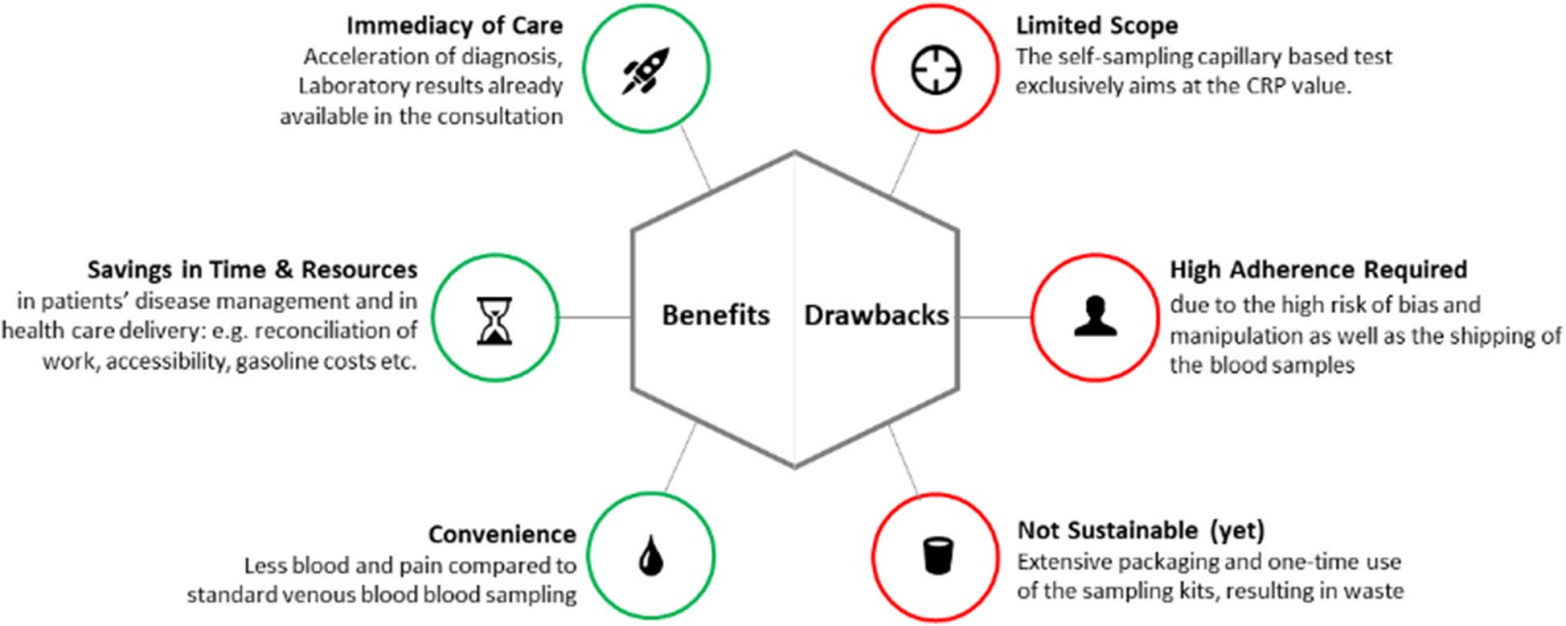
Main benefits and drawbacks of blood self-sampling

Another objective of the present study was to evaluate the perspectives of patients and HCPs on the implementation of capillary blood self-sampling in standard rheumatology care. Below, we present results regarding potential benefits and drawbacks (Fig. 2) as well as necessary requirements for implementation in standard rheumatology care (Table 3).

**Table 3:** Transfer to standard rheumatology care

**Potential benefits of implementation to standard care**
*“Yes, that's a time saver. So with me it's fine to have time off. But for some others, they probably have to take a whole day off. So then it's already a good thing if you can do it yourself.” (Patient 10, Sjögren's syndrome, TAP II: 05:34)*
*“For the patients who have to take blood every week or every two weeks, it might be optimal that they don't have to drive miles to the doctor just to take blood. It would be easier. Especially nowadays with the gas prices…” (Patient 13, Sjögren's syndrome, TAP II: 06:34)*
*“Exactly. I'll say it as it is right now, it would be easier for me to send my blood there instead of me stepping outside with Covid, because I don't have any antibodies.” (Patient 5, Antiphospholipid syndrome, Tasso* + *: 14:13)*
*“Fortunately, I don't mind it anymore, but at the very beginning, when it started, here with the 20 needle pricks and so on, I couldn't see my own blood. (…) Perhaps you can take away a little bit of the fear of such people. Because you know, you don't have to have this syringe in your arm anymore.” (Patient 5, Antiphospholipid syndrome, Tasso* + *: 15:42)*
*“For example, if you don't feel so good or something, then I could do it myself. Well, that's always a hassle. I would have to call first and then see if I can get through by phone and then describe what's going on with me. Then you have to see if you can get an earlier appointment, depending on the situation. So that would be kind of quicker to send that away, then maybe save all the hassle.” (Patient 3, Systemic lupus erythematosus, TAP II: 17:07)*
*“Yes, first and foremost, I think, the acceleration of diagnosis, because if the patient in question has clear symptoms, which are also part of the classification criteria and we can then detect the corresponding antibodies at home, for example, then I would be able to call in the patient more quickly.” (HCP 3: 04:26)*
*“Saving appointments for patients with stable disease, who simply need a follow-up examination… And for others, more frequent blood collection may be necessary, making it easy to intervene ahead of time to avoid potential damage that can be read in [abnormal] laboratory parameters before the ‘spook’ even starts. That might not have been possible before. It definitely has massive advantages there.” (HCP 5: 02:14)*
**Potential drawbacks of implementation to standard rheumatology care**
*“The independence is very, very good. The only thing I wonder: The amount of blood is not very much and when I see the amount that is taken during blood sampling at the medical practice and the amount that is taken with the device trialed, then I believe that it’s very reduced, so to a few parameters probably. I think that you then still have to go to the doctor for other furor something.” (Patient 15, Systemic lupus erythematosus, Tasso* + *: 09:46)*
*“If it is really about this one parameter… I think that if other matters are to be examined which cannot be determined with capillary blood, then it is of course nonsense to say that patients come to the hospital, have their venous blood drawn, and then have capillary blood drawn as well. So I think it really only makes sense if it's very time-consuming for the patient, the university is overloaded and one or two specific parameters really need to be examined or what is possible in the context of such a capillary blood sample.” (HCP 4: 12:49)*
*“So, for instance, one limit is the current availability. That's clear, you just have to see what's available at the moment. The other limit is, of course, the costs. So that's all very limited now, because you're pretty dependent on what you can afford or maybe what you're provided with for individual studies. These are still limitations.” (HCP 2: 13:58)*
*“This waste. But to be honest, I don't see that as a disadvantage, because it's no different in the clinic. We also have butterflies and swabs and so on and so forth. But maybe the patients don't notice that, because they don't necessarily watch. Some of them look away when the blood is being taken, and they are perhaps not so aware of this waste. I don't think that is a disadvantage. As far as the costs of the device itself are concerned, I think they are recovered somewhat by the fact that perhaps on-site appointments including transport, doctor's time, time for the patient and so on can be saved. So I don't see the disadvantages very clearly yet.” (HCP 3: 06:45)*
*“So going to the family doctor is always, for me at least, easier, more convenient and makes more sense to me than getting this package at home and then taking the blood myself.” (Patient 8, Granulomatosis with polyangiitis, TAP II: 12:28)*
**Requirements for transfer to standard rheumatology care**
*“And usually I also have the possibility to discuss my blood. So, if the values are not good, I would like to receive a report and I would like to have the opportunity to discuss it by phone or by e-mail afterwards, or to ask for a discussion, that would be important to me.” (Patient 14, Systemic lupus erythematosus, Tasso* + *: 12:06)*
*“Of course, it is clear that a non-expert can hardly make any sense of positive anti-dsDNA antibodies at first. Therefore, this should certainly not be a stand-alone offer to be able to take any antibodies at home. But, as I said, it should certainly be connected with a medical discussion, an explanatory discussion, whether positive or negative. But I think telemedicine is an important pillar that can and should be implemented.” (HCP 3: 05:55)*
*“I firmly believe in it. Yes, I believe that it is indeed absolutely feasible. Of course, you have to see for which diseases. I think this has to be determined on a very individual basis. There are certain individual patient groups where it makes more sense to at least start with it. But I believe that this is transferable, yes. And that you would also have to get the general practitioners on board, so that they might also participate.” (HCP 2: 07:11)*

Major potential benefits of implementing capillary blood self-sampling in standard rheumatology care reported by patients are time- and cost-savings. Self-sampling enables more insightful face-to-face consultations, as blood results are already available, and at the same time can lead to a reduction in overall hospital visits, which was perceived as a major benefit. Furthermore, reduced travelling costs and time were mentioned as advantages of self-sampling by patients and HCP alike. Concomitantly self-sampling could contribute to a better reconciliation of illness and work. Moreover, a reduced risk of infection by interviewees associated with self-sampling compared to blood collection in the hospital was reported as a benefit. The lower volumes of blood that the devices withdraw, easier collection of blood in patients who usually require multiple attempts with venous blood collection and less pain sensation during blood collection could result in less fear associated with blood collection. Patients and HCPs agreed that self-sampling might also help to better interpret new symptoms and decide if a face-to-face visit is necessary. The reduction of routine in-house appointments for patients who are in disease remission could ease the patient pathway and improve healthcare delivery and thus, might result in more efficient rheumatologic care. Additionally, self-sampling could also help to accelerate diagnosis and improve early detection of disease flares. Overall, HCPs and patients described similar benefits.

A substantial drawback inherent to the devices is the single-use status and relatively small blood volume, potentially limiting the number of laboratory tests and biomarker insights or requiring the use of specific analyzers that utilize blood volumes more effectively. Other drawbacks reported by HCPs and patients alike included the high level of adherence needed by patients to implement self-sampling correctly, connected to risk of bias and manipulation. As described in the "Feasibility" section, both patients and HCPs reported the extensive packaging and single-use of the sampling kits, resulting in a lot of waste, compared to standard blood collection. Furthermore, current low availability of sampling kits and high device costs were mentioned by HCPs as drawbacks. The aspects were opposed by one HCP (HCP 3: 06:45). In addition, few patients reported that it would be easier for them to visit the primary care practice to have blood drawn than to perform self-sampling at home.

The interviewees were asked about necessary requirements for the implementation of self-sampling in standard rheumatology care. A central need expressed by HCPs as well as patients was the possibility for patients to discuss self-sampling results with HCPs. Interviewed HCPs emphasized the importance of involving primary care physicians into self-sampling process. Finally, a thorough assessment was emphasized to determine which patients and which conditions are appropriate for self-sampling.

### Potential user groups

Overall, interviewees considered the majority of rheumatic patients being eligible and able to perform self-sampling (Table 4). Diseases with an established serological biomarker for disease monitoring appear favorable for self-sampling, such as CRP for arthritis patients or anti-dsDNA antibodies for SLE patients. Furthermore, self-sampling could be offered to patients with difficulties accessing medical care, e.g. in rural areas or immobile patients. Potential users should show a high level of adherence, openness for self-management and willingness to independently draw blood. Furthermore, self-sampling might be particularly suitable for patients who are afraid of needles or where a traditional venous blood collection is difficult to carry out. In contrast, patients with temporarily or permanently reduced functional status (i.e. low finger strength, pain) might not be able to successfully collect blood. Furthermore, patients under anticoagulant therapy can be faced with prolonged bleeding. Younger patients were primarily considered to be better suited by patients and HCPs, as they were attributed to generally have less time compared to older patients that do not work anymore and enjoy spending time with their general physician. Yet, age as a criterion for exclusion was questioned by HCPs, as older patients with poor access to venous blood collection sites might also benefit from self-sampling of capillary blood.

**Table 4:** Potential user groups

**Eligibility**
*“But I think one conclusion is definitely that the very majority of patients—I believe about at least nine out of ten patients get along very well with these devices and the blood collection is possible without any problems.” (HCP 1: 18:07)*
*“But especially with older patients who live in the countryside or are no longer so physically mobile, or as is the case now in immunology, with the patients who only go for blood tests every three months, it would be ideal.” (Patient 2, Systemic sclerosis, TAP II: 20:52)*
**Limitations of blood self-sampling**
*“So pressing until it clicks, well, it depends on how much strength you have in your finger or whether you have functional problems or something, then it's a bit difficult to press.” (Patient 11, Systemic lupus erythematosus, TAP II: 03:38)*
*“The only thing with me- I do take blood thinners. Maybe it's different for someone else who doesn't take blood thinners. (…) I would have to squeeze it off somehow.” (Patient 3, Systemic lupus erythematosus, TAP II: 05:38)*
**Age as an indication**
*“For us as older people it is not interesting and for younger people it certainly will be. At some point… the doctors' offices will become fewer and fewer, in the countryside anyway. (…) And for older people, that's already a problem. And then, if you have your family doctor still nearby, you're used to that contact.” (Patient 10, Sjögren's syndrome, TAP II: 03:04)*
*“So it's just a little bit limited, I think, and it's probably not for very old people who are maybe already very entrenched in their routines and for whom going to the family doctor is the most normal thing in the world. And they don't want to miss it. It probably wouldn't be appropriate for them.” (HCP 1: 12:53)*
*“Of course, rather younger people—They get along with it better… But that's not 100% true, because I also had a few participants, who worked in the lab themselves in some way, and with them it went smoothly. I didn't have to explain anything to them. They looked at it once and then they did it in a blink of an eye and it was done, and I could hardly keep up with the documentation, but they did everything right. So clearly, if someone is already working in the medical-technical laboratory area by nature, they had no problems with it at all. And others, where I thought, okay, I would have thought that they could easily do it, they somehow had a bit of a hard time.” (HCP 4: 07:55)*

## Discussion

This qualitative study reports on SRD-patients' and HCPs' experiences and preferences regarding at-home capillary blood self-sampling in rheumatology care. Easy application and high usability were reported. Yet, adverse outcomes and challenges were also expressed, such as poor wound healing and prolonged bleeding. Patients and HCPs alike reported various potential benefits such as time savings, acceleration of disease management, increased independence and flexibility for the patient, which leads to improved reconciliation of disease and work, as well as reduced risk of infection with respiratory diseases during hospital and in-office visits particularly important for immunosuppressed patients. Reported drawbacks of self-sampling are the limited blood volume, ecological footprint, need for certain strength and motoric hand functions, respectively, to activate the lancet. Self-sampling is certainly not a solution that is an appropriate option for all patients and should therefore not aim to replace venous blood collection.

Suitability of self-sampling for the individual patient should be checked by HCPs and openly discussed with patients. Additionally, patients should have direct access to the laboratory results and the opportunity to discuss these with their treating physician. Optimally, the laboratory results from at-home self-sampling and consecutive virtual appointments can guide disease management, including the need for face-to-face visits and treatment changes.

Our results are in line with findings on self-sampling concepts from other medical domains: In diabetes management, self-collection of blood has been extensively studied [33]. Additionally, there is also evidence that self-swabs in screening for sexually transmitted infections are useful tools that support the diagnosis of human papilloma virus [34]. The acceptance of self-sampling was further accelerated due to extensive testing for SARS-CoV 2 infections during the COVID-19 pandemic that was often performed by the patient or at a remote point of care [35], which allows patients easier access to testing and reduces infection risk [36]. Plus, self-collection of specimens for SARS-CoV-2 testing, preparation and shipment of specimens for analysis are well accepted [37].

Our results indicate that, according to users, self-sampling might represent a useful addition to remote rheumatology care for certain patients. Thus, our qualitative data confirm the survey results of Kernder et al. that depicted the willingness of rheumatologists and rheumatic patients to incorporate self-sampling into routine care [9, 38]. However, as in other medical domains, patient empowerment and opportunities for consultation with health care professionals are essential prerequisites for scaling self-sampling to the “new norm” in rheumatology care [39]. Similar to the implementation of telehealth technologies [40], careful consideration must be given in advance to which form of blood collection is appropriate for which patients, in which situation. Thus, we compiled a brief checklist that may guide HCPs in the first assessment of patient suitability (Table 5).

**Table 5:** Checklist for assessing appropriateness of self-sampling

Favorable Factors	Unfavorable Factors
+ Disease with serological biomarker(s) relevant for disease monitoring (SLE, vasculitis, rheumatoid arthritis, spondyloarthritis, psoriatic arthritis)	­ - No clinically relevant serological bio-marker available (i.e. osteoarthritis)
+ Analysis of few biomarkers sufficient (i.e. CRP, anti-dsDNA)	­ - Under anticoagulant therapy
+ Long travel / queue time for venous blood collection	­ - Poor wound healing, eczema and other inflammatory skin disease
+ Limited availability due to work/family responsibilities	­ - Reduced finger strength/function and pain
+ Immobile patient (high effort/burden associated with medical consultation)	­ - Scared of blood self-collection
+ Scared of venous blood collection	­ - Previous failure of blood self-collection
+ Venous blood collection difficult to perform	­ - Limited interest in self-collection
+ Stable disease where main aim of appointment is blood-collection and discussion of blood results could be carried out remotely	
+ Active disease, self-sampling allowing earlier/more frequent analysis of therapeutic response	
+ High patient adherence	

In line with the recent increase of telemedicine usage in rheumatology [9, 41, 42], interviewees acknowledged the potential of self-sampling to enhance current rheumatology remote care, improving accessibility and unlocking resources [43]. Remote self-monitoring, using electronic patient reported outcomes (ePROs), in conjunction with laboratory calprotectin test results that were based on self-collected stool samples improved the management of patients with inflammatory bowel disease [44, 45]. De Thurah et al. could demonstrate that CRP testing together with ePRO-based telehealth follow-up strategy, achieve similar disease control as conventional outpatient follow up, while reducing the number of rheumatologist visits at the same time [18]. Similarly, we are currently investigating the potential of ePROS in combination with semi-quantitative CRP results from point of care in RA [46] and the TAP II devices for precise CRP measurement in patients with spondyloarthritis. In terms of diagnosis, self-sampling could improve the currently limited accuracy of very popular symptom-checkers through consideration of objective laboratory results [47, 48].

Self-sampling might not only have clinical—but also environmental, social and economic impact: Interviewees living in rural areas described that they have to take a whole day off work to visit the hospital for standard venous blood sampling, which results in productivity losses [49] and indirect medical costs [50]. Self-sampling could contribute to a better compatibility of illness and work. Moreover, according to a recent WHO report, the Covid-19 pandemic has led to a massive increase in health care waste [51]. Part of this waste are single-use test kits and personal protective equipment. The results of our study suggest the rising environmental awareness in medical care. Patients criticized the large amount of waste associated with self-sampling and the fact that trialed test kits are single use only. This was contrasted by one interviewee, who indicated that the medical waste would be generated in standard blood-sampling as well, except that the waste remains in the medical institution and is therefore less visible to patients. Nevertheless, self-sampling could reduce the need for personal protective equipment [51] and emissions for trips to the hospital.

To our knowledge, this is the first qualitative study on patients’ and care providers’ perspectives on at-home self-sampling in rheumatology. The qualitative study design allowed for an in-depth description of the participants’ perceptions. Due to the open and exploratory approach patients and HCPs were able to state miscellaneous benefits and drawbacks of self-sampling, as well as their ideas regarding transferability to clinical routine. However, there are certain limitations to the qualitative design of this study. As the 20 interviews do not allow any conclusions regarding the success rate, usability and acceptance of the two trialed devices. These areas require further research, respectively were analyzed separately based on quantitative data [28]. Furthermore, statements of the included participants from a single study site may not be representative, as only patients with some rheumatic diseases and largely female (93%) were included. In addition, only physicians and medical students were included. Interviews with other HCP groups, such as rheumatology nurses, would have contributed to a more comprehensive picture regarding the transferability of blood self-sampling to standard care. The interviewed HCPs were very young, with limited work experience at a university hospital. We nevertheless chose to interview medical students because they supervised the blood self-sampling procedure in this and another study [52]. Recall bias cannot be excluded due to a time difference between self-sampling and the interview. In addition, results may be biased toward the benefits of self-sampling as participants agreed to participate in the study and HCPs were involved in the conduction of the study.

## Conclusion

Self-sampling could be a valuable addition to remote rheumatology care, promising more convenience to patients and improved efficiency of scarce rheumatology services. Limited blood volume, required patients’ adherence and lack of sustainability represent current drawbacks. Individual patient assessment is required to determine the likelihood of successful adoption and extent of the benefit.

## Supplementary Information

**Additional file 1: Supplemental Material 1.** Interview Guide – Patient.

**Additional file 2: Supplemental Material 2.** Interview Guide – HCP.

**Additional file 3: Supplemental Material 3.** Coding Tree.

**Additional file 4.** COREQ (COnsolidated criteria for REporting Qualitative research) Checklist.

## Data Availability

All data relevant to the study are included in the article or uploaded as supplementary information. For further questions regarding the reuse of data, please contact the corresponding author (felix.muehlensiepen@mhb-fontane.de).

## References

[1] Safiri S, Kolahi AA, Cross M, Hill C, Smith E, Carson-Chahhoud K (2021). Prevalence, Deaths, and Disability-Adjusted Life Years Due to Musculoskeletal Disorders for 195 Countries and Territories 1990–2017. Arthritis & rheumatology (Hoboken, NJ).

[2] Branco JC, Rodrigues AM, Gouveia N, Eusébio M, Ramiro S, Machado PM (2016). Prevalence of rheumatic and musculoskeletal diseases and their impact on health-related quality of life, physical function and mental health in Portugal: results from EpiReumaPt- a national health survey. RMD Open.

[3] Xavier RM, Zerbini CAF, Pollak DF, Morales-Torres JLA, Chalem P, Restrepo JFM (2019). Burden of rheumatoid arthritis on patients’ work productivity and quality of life. Advances in Rheumatology.

[4] Orbai AM, Reddy SM, Dennis N, Villacorta R, Peterson S, Mesana L (2021). Work absenteeism and disability associated with psoriasis and psoriatic arthritis in the USA—a retrospective study of claims data from 2009 TO 2020. Clin Rheumatol.

[5] Deal CL, Hooker R, Harrington T, Birnbaum N, Hogan P, Bouchery E (2007). The United States rheumatology workforce: supply and demand, 2005–2025. Arthritis Rheum.

[6] Xiang L, Low AHL, Leung YY, Fong W, Gandhi M, Yoon S (2021). Interval between symptom onset and diagnosis among patients with autoimmune rheumatic diseases in a multi-ethnic Asian population. Int J Rheum Dis.

[7] van der Linden MP, le Cessie S, Raza K, van der Woude D, Knevel R, Huizinga TW (2010). Long-term impact of delay in assessment of patients with early arthritis. Arthritis Rheum.

[8] Dejaco C, Alunno A, Bijlsma JW, Boonen A, Combe B, Finckh A (2021). Influence of COVID-19 pandemic on decisions for the management of people with inflammatory rheumatic and musculoskeletal diseases: a survey among EULAR countries. Ann Rheum Dis.

[9] Kernder A, Morf H, Klemm P, Vossen D, Haase I, Mucke J (2021). Digital rheumatology in the era of COVID-19: results of a national patient and physician survey. RMD open.

[10] Nikiphorou E, Santos EJF, Marques A, Böhm P, Bijlsma JW, Daien CI, et al. 2021 EULAR recommendations for the implementation of self-management strategies in patients with inflammatory arthritis. Ann Rheum Dis. 2021;80(10):1278–85. 10.1136/annrheumdis-2021-220249 PMC8458093 33962964

[11] McDougall JA, Ferucci ED, Glover J, Fraenkel L (2017). Telerheumatology: A Systematic Review. Arthritis Care Res.

[12] Proft F, Spiller L, Redeker I, Protopopov M, Rodriguez VR, Muche B (2020). Comparison of an online self-referral tool with a physician-based referral strategy for early recognition of patients with a high probability of axial spa. Semin Arthritis Rheum.

[13] Knitza J, Mohn J, Bergmann C, Kampylafka E, Hagen M, Bohr D (2021). Accuracy, patient-perceived usability, and acceptance of two symptom checkers (Ada and Rheport) in rheumatology: interim results from a randomized controlled crossover trial. Arthritis Res Ther.

[14] Knitza J, Muehlensiepen F, Ignatyev Y, Fuchs F, Mohn J, Simon D (2022). Patient's Perception of Digital Symptom Assessment Technologies in Rheumatology: Results From a Multicentre Study Frontiers in Public. Health.

[15] Müskens  WD, Rongen-van Dartel SAA, Vogel C, Huis A, Adang EMM, van Riel P (2021). Telemedicine in the management of rheumatoid arthritis: maintaining disease control with less health-care utilization. Rheumatology advances in practice.

[16] Hendrikx J, Fransen J, van Riel PL (2015). Monitoring rheumatoid arthritis using an algorithm based on patient-reported outcome measures: a first step towards personalised healthcare. RMD Open.

[17] Skovsgaard CV, Kruse M, Hjollund N, Maribo T, de Thurah A. Cost-effectiveness of a telehealth intervention in rheumatoid arthritis: economic evaluation of the Telehealth in RA (TeRA) randomized controlled trial. Scand J Rheumatol. 2022. 10.1080/03009742.2021.2008604. 10.1080/03009742.2021.2008604 35048793

[18] de Thurah A, Stengaard-Pedersen K, Axelsen M, Fredberg U, Schougaard LMV, Hjollund NHI (2018). Tele-Health Followup Strategy for Tight Control of Disease Activity in Rheumatoid Arthritis: Results of a Randomized Controlled Trial. Arthritis Care Res.

[19] Heneghan CJ, Garcia-Alamino JM, Spencer EA. Self‐monitoring and self‐management of oral anticoagulation. Cochrane Database Syst Rev. 2016;(7):CD003839. 10.1002/14651858.CD003839.pub3 PMC8078378 27378324

[20] Lin R, Brown F, James S, Jones J, Ekinci E (2021). Continuous glucose monitoring: A review of the evidence in type 1 and 2 diabetes mellitus. Diabetic medicine: a journal of the British Diabetic Association.

[21] Kneepkens EL, Pouw MF, Wolbink GJ, Schaap T, Nurmohamed MT, de Vries A (2017). Dried blood spots from finger prick facilitate therapeutic drug monitoring of adalimumab and anti-adalimumab in patients with inflammatory diseases. Br J Clin Pharmacol.

[22] Qu Y, Brady K, Apilado R, O'Malley T, Reddy S, Chitkara P (2017). Capillary blood collected on volumetric absorptive microsampling (VAMS) device for monitoring hydroxychloroquine in rheumatoid arthritis patients. J Pharm Biomed Anal.

[23] Orange DE, Yao V, Sawicka K, Fak J, Frank MO, Parveen S (2020). RNA Identification of PRIME Cells Predicting Rheumatoid Arthritis Flares.

[24] Hendelman T, Chaudhary A, LeClair AC, van Leuven K, Chee J (2021). Self-collection of capillary blood using Tasso-SST devices for Anti-SARS-CoV-2 IgG antibody testing. PLoS ONE.

[25] Solheim SA, Ringsted TK, Nordsborg NB, Dehnes Y, Levernaes MCS, Mørkeberg J (2021). No pain, just gain: Painless, easy, and fast dried blood spot collection from fingertip and upper arm in doping control. Drug Test Anal.

[26] Blicharz TM, Gong P, Bunner BM, Chu LL, Leonard KM, Wakefield JA (2018). Microneedle-based device for the one-step painless collection of capillary blood samples. Nature biomedical engineering.

[27] Knitza J, Tascilar K, Vuillerme N, Vogt E, Matusewicz P (2022). Accuracy and tolerability of self-sampling of capillary blood for analysis of inflammation and autoantibodies in rheumatoid arthritis patients-results from a randomized controlled trial. Arthritis Res Ther.

[28] Zarbl J, Eimer E, Gigg C, Bendzuck G, Korinth M, Elling-Audersch C (2022). Remote self-collection of capillary blood using upper arm devices for autoantibody analysis in patients with immune-mediated inflammatory rheumatic diseases. RMD Open.

[29] Etikan I, Musa SM, Alkassim RS (2016). Comparison of Convenience Sampling and Purposive Sampling. Am J Theor Appl Stat.

[30] Kuckartz U (2018). Qualitative Inhaltsanalyse.

[31] Hennink MM, Kaiser BN, Marconi VC (2017). Code Saturation Versus Meaning Saturation: How Many Interviews Are Enough Qualitative Health Research.

[32] Tong A, Sainsbury P, Craig J (2007). Consolidated criteria for reporting qualitative research (COREQ): a 32-item checklist for interviews and focus groups. International journal for quality in health care: journal of the International Society for Quality in Health Care.

[33] Malanda UL, Welschen LM, Riphagen II, Dekker JM, Nijpels G, Bot SD (2012). Self-monitoring of blood glucose in patients with type 2 diabetes mellitus who are not using insulin. The Cochrane database of systematic reviews.

[34] Yeh PT, Kennedy CE, de Vuyst H, Narasimhan M (2019). Self-sampling for human papillomavirus (HPV) testing: a systematic review and meta-analysis. BMJ Glob Health.

[35] Tsang NNY, So HC, Ng KY, Cowling BJ, Leung GM, Ip DKM (2021). Diagnostic performance of different sampling approaches for SARS-CoV-2 RT-PCR testing: a systematic review and meta-analysis. Lancet Infect Dis.

[36] Wehrhahn MC, Robson J, Brown S (2020). Self-collection: An appropriate alternative during the SARS-CoV-2 pandemic. J Clin Virol.

[37] Valentine-Graves M, Hall E, Guest JL (2020). At-home self-collection of saliva, oropharyngeal swabs and dried blood spots for SARS-CoV-2 diagnosis and serology: Post-collection acceptability of specimen collection process and patient confidence in specimens. PLoS ONE.

[38] Morf H, Krusche M, Knitza J (2021). Patient self-sampling: a cornerstone of future rheumatology care?. Rheumatol Int.

[39] Boum Y, Eyangoh S, Okomo M-C (2021). Beyond COVID-19-will self-sampling and testing become the norm?. Lancet Infect Dis.

[40] Kulcsar Z, Albert D, Ercolano E, Mecchella JN (2016). Telerheumatology: A technology appropriate for virtually all. Semin Arthritis Rheum.

[41] Muehlensiepen F, Knitza J, Marquardt W, Engler J, Hueber A, Welcker M (2021). Acceptance of Telerheumatology by Rheumatologists and General Practitioners in Germany: Nationwide Cross-sectional Survey Study. J Med Internet Res.

[42] Muehlensiepen F, Knitza J, Marquardt W, May S, Krusche M, Hueber A (2021). Opportunities and Barriers of Telemedicine in Rheumatology: A Participatory, Mixed-Methods Study. Int J Environ Res Public Health.

[43] Mühlensiepen F, Kurkowski S, Krusche M, Mucke J, Prill R, Heinze M (2021). Digital Health Transition in Rheumatology: A Qualitative Study. Int J Environ Res Public Health.

[44] Pedersen N, Thielsen P, Martinsen L, Bennedsen M, Haaber A, Langholz E (2014). eHealth: individualization of mesalazine treatment through a self-managed web-based solution in mild-to-moderate ulcerative colitis. Inflamm Bowel Dis.

[45] Pedersen N, Elkjaer M, Duricova D, Burisch J, Dobrzanski C, Andersen NN (2012). eHealth: individualisation of infliximab treatment and disease course via a self-managed web-based solution in Crohn’s disease. Aliment Pharmacol Ther.

[46] Mucke J, Knitza J, Muehlensiepen F, Grahammer M, Stenzel R, Simon D, et al. TELERA—Asynchronous TELEmedicine for Patients With Rheumatoid Arthritis: Study Protocol for a Prospective, Multi-Center, Randomized Controlled Trial. Front Med Rheumatol. 2021;8. 10.3389/fmed.2021.791715. 10.3389/fmed.2021.791715 PMC8710736 34966765

[47] Knitza J, Knevel R, Raza K, Bruce T, Eimer E, Gehring I (2020). Toward Earlier Diagnosis Using Combined eHealth Tools in Rheumatology: The Joint Pain Assessment Scoring Tool (JPAST) Project. JMIR Mhealth Uhealth.

[48] Knitza J, Tascilar K, Gruber E, Kaletta H, Hagen M, Liphardt A-M (2021). Accuracy and usability of a diagnostic decision support system in the diagnosis of three representative rheumatic diseases: a randomized controlled trial among medical students. Arthritis Res Ther.

[49] Neovius M, Simard JF, Askling J (2011). ARTIS Study Group How large are the productivity losses in contemporary patients with RA, and how soon in relation to diagnosis do they develop?. Ann Rheum Dis.

[50] Raciborski F, Kłak A, Kwiatkowska B (2015). Indirect costs of rheumatoid arthritis. Reumatologia.

[51] World Health Organization. Global analysis of health care waste in the context of COVID-19. Status, impacts and recommendations. 1st edn. Genf. 2022. https://www.who.int/publications/i/item/9789240039612. Accessed 15 Nov 2022.

[52] Schmetzer C, Vogt E, Stellar L, Godonou E-T, Liphardt A-M, Muehlensiepen F, et al. Self-collection of capillary blood and saliva to determine COVID-19 vaccine immunogenicity in patients with immune-mediated inflammatory diseases and health professionals. Front Public Health. 2022;10. 10.3389/fpubh.2022.994770. 10.3389/fpubh.2022.994770 PMC9616117 36311633

