# Supplementary material for: At-home blood self-sampling in rheumatology: a qualitative study with patients and health care professionals
Source: BMC Health Serv Res. 2022 Dec 2;22:1470. doi: 10.1186/s12913-022-08787-5 (PMC9718468; doi:10.1186/s12913-022-08787-5)
Supplement: Supplementary file 1 — Additional file 1: Supplemental Material 1. Interview Guide – Patient. [file 12913_2022_8787_MOESM1_ESM.docx]

**Supplemental Material 1: Interview Guide – Patient**

| Guiding question/ narrative stimulus | Check aspects | Concretizing questions | | Control questions |
| --- | --- | --- | --- | --- |
| You are participating in a study in which you are sampling blood on your own.  Can you please tell me how this all came about?  Please start at the doctor's visit when you were asked if you would like to participate? | Experience of the intervention  Differences to regular care | - How did your doctor(s) address the study?  - Please tell us what went through your mind...  - ...for what reasons you decided to participate in the study?  - What came after? Did you receive the material and then did you draw your own blood? | | Can you tell me more about this?  And then?  What was that like for you?  How do you perceive this?  Can you elaborate on that, please?  Could you give an example of that, please?  What do you mean specifically?  Can you tell me more about this?  And then?  What was that like for you?  How do you perceive this?  Can you elaborate on that, please?  Could you give an example of that, please?  What do you mean specifically?  Can you tell me more about that?  And then?  What was that like for you?  How do you see that? Can you elaborate on that, please?  Could you give an example of that, please?  What do you mean specifically? |
| Please tell me about the first time you drew your own blood.  What was that like? | Acceptance  User experience  User behaviour  Usability  Handling  Visual, haptic aspects  Comparison  Expectations  Effort | - Did you know directly what to do?  - Did you have any questions? [Information sheet / call: practice?]  - Were you excited?  [Description and follow-up questions about each step]  - How did it work at home?  - Did everything work out? [If no, ask in more detail]  - Did it hurt?  - What did you do next?  - Did the blood draw meet your expectations and what you were told in the [organizational unit]?  - Is there anything you would improve about the whole procedure? ...or what you would prefer differently? | |  |
| Could you imagine collecting blood samples at home more often in the future? | Autonomous blood collection  Benefits  Drawbacks | - What would that depend on? Would there be any prerequisites?  - What would be important to you? (e.g. support, telephone contact persons)  - When you think about the standard blood draw, which mode do you prefer? The way it used to be or drawing blood yourself from home?  - What potential drawbacks do you perceive? (compared to standard care?)  - What potential benefits do you perceive?  - Is there anything else you noticed?  - Is there anything else you would like to address? | |  |
| **Age** | | | **Gender** | |
|  | | |  | |
| **Diagnosis** | | | **Profession** | |
|  | | |  | |
