# Supplementary material for: At-home blood self-sampling in rheumatology: a qualitative study with patients and health care professionals
Source: BMC Health Serv Res. 2022 Dec 2;22:1470. doi: 10.1186/s12913-022-08787-5 (PMC9718468; doi:10.1186/s12913-022-08787-5)
Supplement: Supplementary file 2 — Additional file 2: Supplemental Material 2. Interview Guide – HCP. [file 12913_2022_8787_MOESM2_ESM.docx]

**Supplemental Material 2: Interview Guide – HCP**

| Guiding question/ narrative stimulus | Check aspects | Concretizing questions | | Control questions |
| --- | --- | --- | --- | --- |
| Your [organizational unit] is participating in a study in which patients are taking blood samples on their own.  Could you please describe your experiences to me? | Experiencing the intervention  Adverse events  Benefits  Drawbacks  Hurdles  Opportunities  Target groups | - What benefits do you perceive in patients being able to draw blood independently at home?  - What Drawbacks do you perceive?  - Where do you see barriers?  - Was the blood collection painful?  - What helps with the implementation?  - Did you know before the study that many parameters (antibodies, drug levels) can already be determined using capillary blood? | | Can you tell me more about this?  And then?  What was that like for you?  How do you perceive this?  Can you elaborate on that, please?  Could you give an example of that, please?  What do you mean specifically?  Can you tell me more about this?  And then?  What was that like for you?  How do you perceive this?  Can you elaborate on that, please?  Could you give an example of that, please?  What do you mean specifically? |
| The intervention has now been tested in a study.  What is your assessment: is the independent blood sampling transferable to standard care? | Acceptance  Target Groups  Transferability to standard care  Expectations  Comparison: Standard care  Potential | - What would that depend on? Would there be any prerequisites?  - Which patients are suitable for at home self-sampling?  - Which ones are not?  - Did the study meet your expectations and what you were told in the [organizational unit]?  - Is there anything you would improve about the whole procedure? ...or anything you would prefer to be done differently?  - Are there limits to autonomous blood sampling? If so, what are they?  - Is there anything else you noticed?  - Is there anything else you would like to address? | |  |
| **Age** | | | **Gender** | |
|  | | |  | |
| **Profession** | | | **Notes** | |
|  | | |  | |
