# Supplementary material for: At-home blood self-sampling in rheumatology: a qualitative study with patients and health care professionals
Source: BMC Health Serv Res. 2022 Dec 2;22:1470. doi: 10.1186/s12913-022-08787-5 (PMC9718468; doi:10.1186/s12913-022-08787-5)
Supplement: Supplementary file 3 — Additional file 3: Supplemental Material 3. Coding Tree. [file 12913_2022_8787_MOESM3_ESM.docx]

**Supplemental Material 3: Coding Tree**

| **Main Category** | **Category** | **Group** | **Code (Examples)** |
| --- | --- | --- | --- |
| Feasibility | Description of the self-sampling process | Patient | *(No subcodes assigned)* |
|  | Positive experiences | Patient | Smooth application, easy usage, high success rate |
|  | Negative experiences | Patient | Environmental issues, persisting wounds and hemorrhage, hard to press button to initiate blood collection, hard to remove device |
| Transfer to standard rheumatology care | Benefits of implementation to standard rheumatology care | Patient | Convenience, independence, cost- and time-savings, reduction of unnecessary appointments, reduced risk of infection, lower number of blood collection attempts |
|  |  | HCP | Acceleration of diagnosis, access to more frequent laboratory results, earlier detection of disease flares |
|  | Drawbacks of implementation to standard rheumatology care | Patient | Single use device producing a lot of waste, easy access to primary care blood collection |
|  |  | HCP | Limited scope of test due to low blood volume |
|  | Requirements for transfer to rheumatology standard care | Patient | Contact person for device usage |
|  |  | HCP | Include primary care |
| Potential user groups | Eligibility | Patient | Difficulties accessing medical care, high adherence |
|  |  | HCP | Diseases with an established serological biomarker |
|  | Limitations | Patient | Patients with reduced functional status |
|  |  | HCP | Under anticoagulant therapy |
|  | Age as an indication | Patient | *(No subcodes assigned)* |
|  |  | HCP |  |
